# Gut bacterial metabolite Urolithin A inhibits myocardial fibrosis through activation of Nrf2 pathway in vitro and in vivo

**DOI:** 10.1186/s10020-022-00444-1

**Published:** 2022-02-08

**Authors:** Pengfei Chen, Junyu Pei, Xiaopu Wang, Shi Tai, Liang Tang, Xinqun Hu

**Affiliations:** Department of Cardiology, The Second Xiangya Hospital of Central South University, No.139, Middle Ren-min Road, Changsha, 410011 Hunan Province People’s Republic of China

**Keywords:** Myocardial fibrosis, Urolithin A, Myofibroblast transformation, Nrf2

## Abstract

**Background:**

Myocardial fibrosis after myocardial infarction (MI) is one of the leading causes of cardiovascular diseases. Cardiac fibroblasts (CFs) are activated and promoted by MI to undergo myofibroblast transformation (CMT). Urolithin A (UA) is an active and effective gut metabolite derived from polyphenolics of berries and pomegranate fruits, which has been reported to have anti-inflammatory and anti-oxidant functions. However, whether UA affects the CMT process during myocardial fibrosis remains unclear.

**Methods:**

TGF-β1-treated primary rat cardiac fibroblasts were used for in vitro study. Cell proliferation ability was evaluated by MTT assay. Cell migration and invasion abilities were tested by wound healing and Transwell assays. The expression of CMT process-related markers were measured by qRT-PCR and western blot. The rat MI model was established by left anterior descending coronary artery (LAD) ligation and evaluated by H&E and Masson staining.

**Results:**

Our data demonstrated that UA treatment could inhibit the CMT process in TGF-β1-induced CFs, including cell proliferation, migration and invasion abilities. Knocking down of Nrf2, which was activated by UA treatment, could mitigate the effects of UA treatment on CMT process. Moreover, in vivo administration of UA in rat MI model successfully up-regulated Nrf2 expression and improved the myocardial damage and fibrosis.

**Conclusions:**

The study discovered the function and mechanism of UA on myocardial fibrosis and demonstrated the protective effects of UA administration through activation of Nrf2 pathway.

## Introduction

Myocardial fibrosis is one of the most important pathological changes after myocardial infarction (MI), which is the main cause of sudden death around the world (Chen et al. 2013). Myocardial fibrosis could result in ventricular dilation, infarct expansion and heart failure (Gajarsa et al. 2011). Consequently, inhibition of myocardial fibrosis after MI is critical for the recovery of heart injury and reduction of mortality.

During the recent years, more and more studies have focused on the new strategies against myocardial fibrosis through modulating myocardial microenvironment. Specifically, different dietary polyphenols have attracted attentions due to their anti-inflammatory and anti-oxidant properties (Koch 2019). Urolithin A (UA, 3,8-dihydroxy-urolithin) is a kind of metabolite detected in human biological fluids and tissues, produced by gut microbiota from ellagitannin (ET) and ellagic acid (EA), complex polyphenols mainly present in strawberries, pomegranates, walnuts and raspberries (Espin et al. 2013). UA was first identified as an EA metabolite in rats and then demonstrated to be transformation from ET across many species, including human gut microbiota (Cerda et al. 2005). Study has shown that UA is produced in the gut starting with tetrahydroxy-urolithin through the removal of one of the lactone rings of EA, following by sequential removal of hydroxyls to end with UA (Espin et al. 2013). UA could circulate in plasma and be further processed as glucuronide and sulfate conjugates at a concentration from 0.2 to 20 μM (Avila-Galvez et al. 2019). Evidences have shown that free availability of UA in inflammatory microenvironmental sites has beneficial effects on bowel diseases or colon cancer (Avila-Galvez et al. 2019; Piwowarski et al. 2017). Meanwhile, UA metabolites have been demonstrated to have anti-aging property by inducing mitophagy (Ryu et al. 2016). Furthermore, a recent study has proved that in vivo UA administration might be helpful for the heart injury recovery by blocking the inflammatory response of myocardial tissues and preventing adverse influence of the altered diabetic milieu on cardiac performance (Savi et al. 2017). However, the molecular mechanism regarding of UA’s function in myocardial fibrosis remains unclear.

The nuclear factor erythroid 2-related factor 2 (Nrf2) is a kind of leucine zipper transcription factor that is mainly expressed in the cytoplasm (Jaramillo et al. 2013). Studies have shown that the accumulation and activation of Nrf2 are favored in oxidative injury to bind antioxidant response elements (AREs), leading to the activation of downstream targets related to antioxidant defense, such as heme oxygenase-1 (HO1) and NAD(P)H dehydrogenase [quinone] 1 (NQO1) (Ahmed et al. 2017; Kavian et al. 2018; Nguyen et al. 2009; Shelton et al. 2013). Due to its function in modulating cellular oxidative stress, Nrf2 has been linked to the cardiac dysfunction and maladaptive cardiac remodeling (Seddon et al. 2007). Nrf2 knockout mice have exhibited a dysfunction of left ventricular diastolic due to cardiac hypertrophy (Erkens et al. 2015).

Intriguingly, evidence has shown that UA could exert its anti-inflammation function through activation of Nrf2 pathway in epithelium cells (Singh et al. 2019). So we speculated that UA’s treatment in injured heart against cardiac fibrosis was also via targeting Nrf2 pathway. In the present study, we examined that function of UA in TGF-β1-treated cardiac fibroblasts and found it be able to inhibit the transformation of cardiac fibroblast to myofibroblast by activating Nrf2 pathway. Furthermore, in vivo administration of UA significantly mitigated myocardia fibrosis in rat myocardial infarction model.

## Methods and materials

### Isolation and culture of primary rat cardiac fibroblasts

All animal experiments were conducted according to the guidelines and approved protocols of the Animal Care and Use Committee of the Second Xiangya Hospital of Central South University. Primary rat cardiac fibroblasts (CFs) were isolated from Sprague–Dawley rats of 1–3 days old using the protocol as previously described (Nemir et al. 2014). Cells were then cultured in Dulbecco’s modified Eagle’s medium (DMEM) supplemented with 10% fetal bovine serum (FBS) in a humidified atmosphere in 5% CO_2_ at 37 °C. All the cells used in this study were no more than three passages.

### Cell treatment and transfection

TGF-β1 (10 ng/mL, R&D, USA) and Urolithin A (Santa Cruz Biotechnology, USA) with indicated concentrations were used to treat cardiac fibroblasts. Negative control siRNA (si-NC) and Nrf2 specific siRNA (si-Nrf2) were designed and purchased from GenePharma (Shanghai, China). Cell transfection was performed by Lipofectamine 3000 (Invitrogen, USA) for 48 h.

### Total RNA isolation and quantitative real-time PCR (qRT-PCR)

Cells and cardiac tissues from rats were dissolved in TRIzol reagent (Cat. 15596018, Invitrogen, USA). Total RNA was obtained according to the manufacturer’s protocol. The RNA was then tested for quality and synthesized into cDNA using an iScript cDNA Synthesis Kit (Cat. 1708891, Bio-Rad, USA). qRT-PCR was performed using iQ™ SYBR Green Supermix (Cat. 1708882, Bio-Rad, USA). GAPDH and U6 were used as endogenous controls for normalization. Relative expression levels were normalized and analyzed using the 2^−ΔΔCt^ method.

### Western blot analysis

Cells were washed with cold PBS and incubated with lysis buffer on ice for 30 min. Then, the cells were scraped, and after centrifugation, the supernatant containing the lysate was collected and stored at − 80 °C. Cardiac tissues from rats were homogenized and then incubated with lysis buffer on ice for 30 min. A BCA assay Kit (Cat. 5000001, Bio-Rad, USA) was used to determine protein concentration. Protein samples were denatured and then separated by SDS-PAGE and transferred to PVDF membranes (Cat. IPVH00010, Millipore, USA). After blocking with non-fat milk for 1 h, membranes were incubated overnight at 4 °C with the following primary antibodies from Cell Signaling Technology (Danvers, USA): α-SMA (#19245), vimentin (#5741), DDR2 (#12133), tensin (#11990), Nrf2 (#12721), SOD1 (#37385), HO-1 (#82206), NQO-1 (#62262) and β-actin (#3700), and all were used at a 1:1000 dilution. After washing 3 times, the membranes were incubated with goat anti-mouse (#7076) or anti-rabbit (#7077) HRP-conjugated secondary antibodies (Cell Signaling Technology, USA). The signals were analyzed using an ECL detection Kit (Cat. 32106, Pierce Biotechnology, USA).

### Cell viability assay

The 3-(4, 5-dimethylthiazol-2-yl)-2,5-diphenyl tetrazolium bromide (MTT) assay (ab211091, Abcam, Cambridge, UK) was used to detect cell viability according to the manufacture’s protocol. Briefly, after indicated treatments, 1 × 10^4^ cells were collected and seeded into 96-well plates and 20 µL of MTT solution was added to each well. After 2 h cultures, the MTT solution was replaced with 200 μL of DMSO. The absorbance at 490 nm was measured using a spectrophotometer.

### Wound healing assay

The protocol was carried out as previously described (Rezabakhsh et al. 2019). Briefly, CFs were seeded with the indicated treatments. After 48 h culturing, the attached cells were scratched by a pipette tip, and images were captured under a microscope immediately after the scratch as 0 h. The plates were then cultured for 24 h. Then, another set of images of the same wounds were captured. The wound area was measured with ImageJ and was normalized and presented as a percentage of the initial wound measured at 0 h.

### Transwell assay

A Transwell assay was performed according to a reported protocol (Matluobi et al. 2018). After the indicated treatments, a total of 5 × 10^5^ CFs were suspended in serum-free culture medium and seeded into the upper insert of a 12-well Transwell plate (Cat. 3401, Corning Incorporated, USA) with matrigel pre-treatment. The lower chamber was filled with medium with serum. The plate was incubated in the incubator for 8 h. Cells remaining in the upper insert were removed using cotton swabs, and the invasive cells were fixed with 4% paraformaldehyde for 10 min. After washing with PBS for 3 times, the cells were stained with a crystal violet solution and imaged using brightfield microscopy (Olympus, Tokyo, Japan) and quantified.

### Rat myocardial infarction model

Myocardial infarction model was established in 8-week old male Sprague–Dawley rats (weighed around 250 ~ 280 g, SJA Laboratory Animal Company, Hunan, China) using left anterior descending coronary artery (LAD) ligation method as described previously (Nemir et al. 2014). Briefly, a 6–0 silk suture slipknot was placed at the proximal one third of LAD. The ligation was accomplished at the same segment of LAD, and then, the anterior wall of left ventricle (LV) turned pale. The same surgical protocols were performed in the sham group except that the suture placed under the left coronary artery was not tied. There were totally 28 rats in sham surgery and 28 rats for MI surgery that further divided into four groups with 14 rats per group: sham, sham plus UA administration, MI, and MI plus UA administration. UA was administrated 24 h after surgery, through daily intraperitoneal injection at a dose of 2.5 mg/kg/day for 7 days (Savi et al. 2017). Equal volume of normal saline was used as negative control for MI and sham groups. After UA administration for 7 days, the heart tissues along the short-axis plane at the level of 1/2 of the distance from the atrioventricular ring to the apex were collected for further analyses. The survival rate of rats in MI groups and sham groups is 85.7% and 100%, respectively. All the surgeries were done by one person to avoid the operation differences. The animal research was permitted via the Ethics Committee of the Second Xiangya Hospital of Central South University.

### Histopathology

Rat heart tissues were fixed in 4% paraformaldehyde solution and embedded in paraffin. After slicing into section (5 μm thick), the sections were dewaxed in xylene and rehydrated in a descending series of alcohol. Slides were then stained with hematoxylin and eosin (H&E) or Masson’s trichrome (Sigma-Aldrich; St. Louis, MO) according to the manufacture’s protocol.

For H&E staining, the myocardial injury was scored according to the following standard: Score 0: no myocardial structure injury; Score 1: slight myocardial mesenchymal edema, gap increase, and local necrosis; Score 2: extensive myocardial swell and mesenchymal edema, and medial local necrosis; Score 3: severe small vessel damage and myocardial necrosis, massive inflammatory cellular infiltration, and formation of contraction bands; Score 4: severe diffusive myocardial necrosis and hemorrhage, accompanied by massive small vessel damage and formation of contraction bands.

For Masson’s trichrome staining, the extent of fibrosis was expressed as the relative fibrosis area, which equals the ratio of Masson’s trichrome-stained area to the whole of myocardial area in view.

### Statistical analysis

Statistical analysis was performed using GraphPad Prism 5. All experiments were conducted at least three times. All data are presented as the mean ± standard deviation (SD. The data were analyzed by one-way analysis of variance (ANOVA) followed by Tukey post hoc test for multiple comparison. P < 0.05 was considered statistically significant.

## Results

### UA inhibited TGF-β1-induced proliferation, migration and invasion of cardiac fibroblasts

**Fig. 1:**
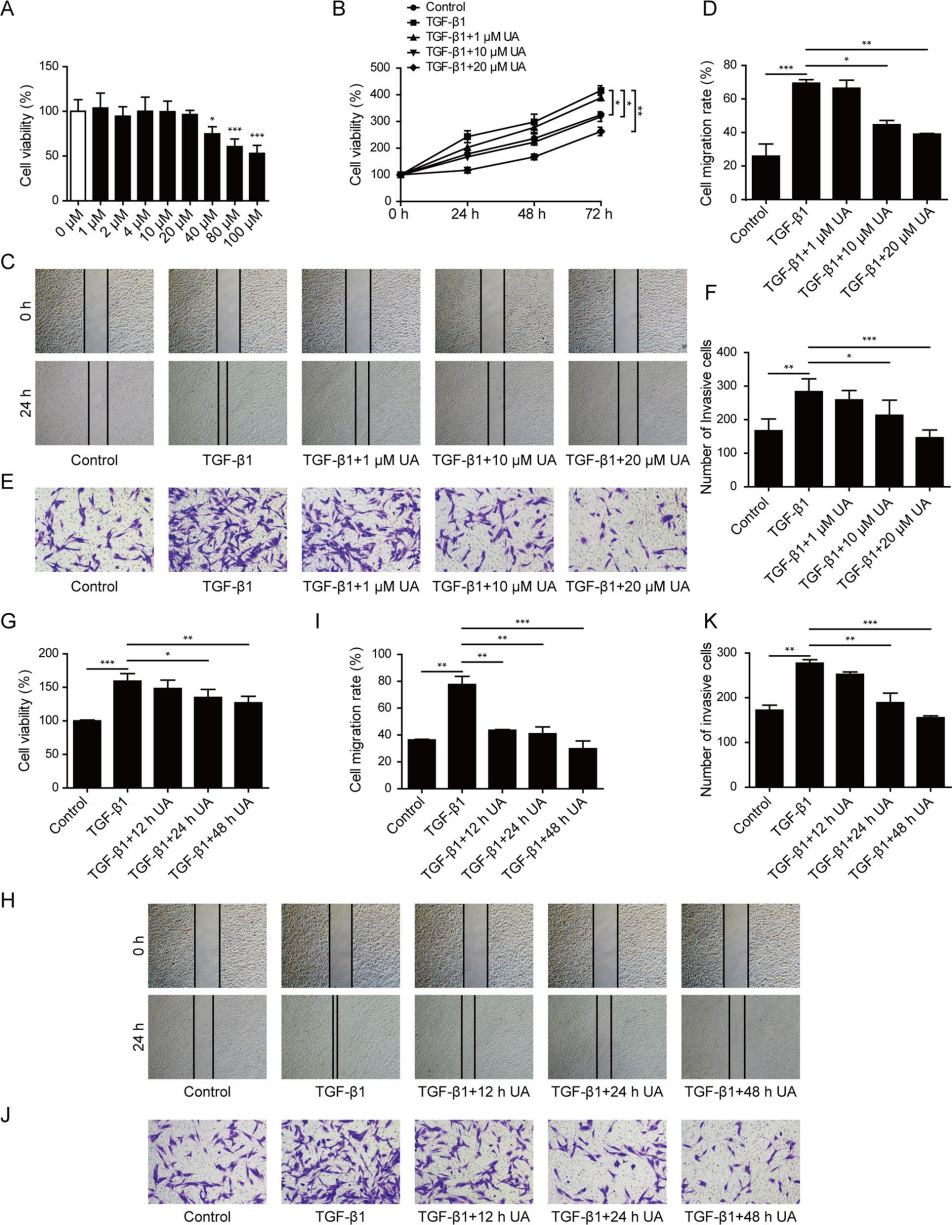
UA inhibited TGF-β1-induced proliferation, migration and invasion of cardiac fibroblasts. **A**, **B** Cell viability of CFs was measured by MTT assay after indicated treatments. **C**, **D** Cell migration was measured by wound healing assay of CFs after indicated treatments. **E**, **F** Cell invasion ability of CFs was measured by transwell assay after indicated treatments. **G** Cell viability of CFs was measured by MTT assay after indicated treatments. **H**, **I** Cell migration was measured by wound healing assay of CFs after indicated treatments. **J**, **K** Cell invasion ability of CFs was measured by transwell assay after indicated treatments. N = 3, *P < 0.05, **P < 0.01, ***P < 0.001

To test the effects of UA on myocardial fibrosis, primary rat cardiac fibroblasts were used as the model for in vitro study. Dose evaluation of UA was firstly determined and the UA (≤ 20 μM) exhibited no effect on cell viability of CFs (Fig. 1A), thus 1, 10, and 20 μM UA were chose to perform the further experiments. As shown in Fig. 1B–F, TGF-β1 (10 ng/mL) treatment significantly increased cell proliferation, migration and invasion of CFs compared to control treatment. When UA was combined with TGF-β1 treatment, it remarkably slowed down cell proliferation rate (Fig. 1B) and inhibited the migration (Fig. 1C–D) and invasion (Fig. 1E–F) abilities of CFs in a dose-dependent manner. Moreover, with a concentration of UA (10 μM) combined with TGF-β1 treatment, cell proliferation (Fig. 1G), migration (Fig. 1H–I) and invasion (Fig. 1J–K) abilities were also gradually decreased from 12 to 48 h. Taken together, these data suggested that UA treatment could counteract TGF-β1-induced proliferation, migration and invasion of cardiac fibroblasts in dose- and time-dependent manners.

### UA treatment repressed TGF-β1-induced transformation of cardiac fibroblasts to myofibroblasts

**Fig. 2:**
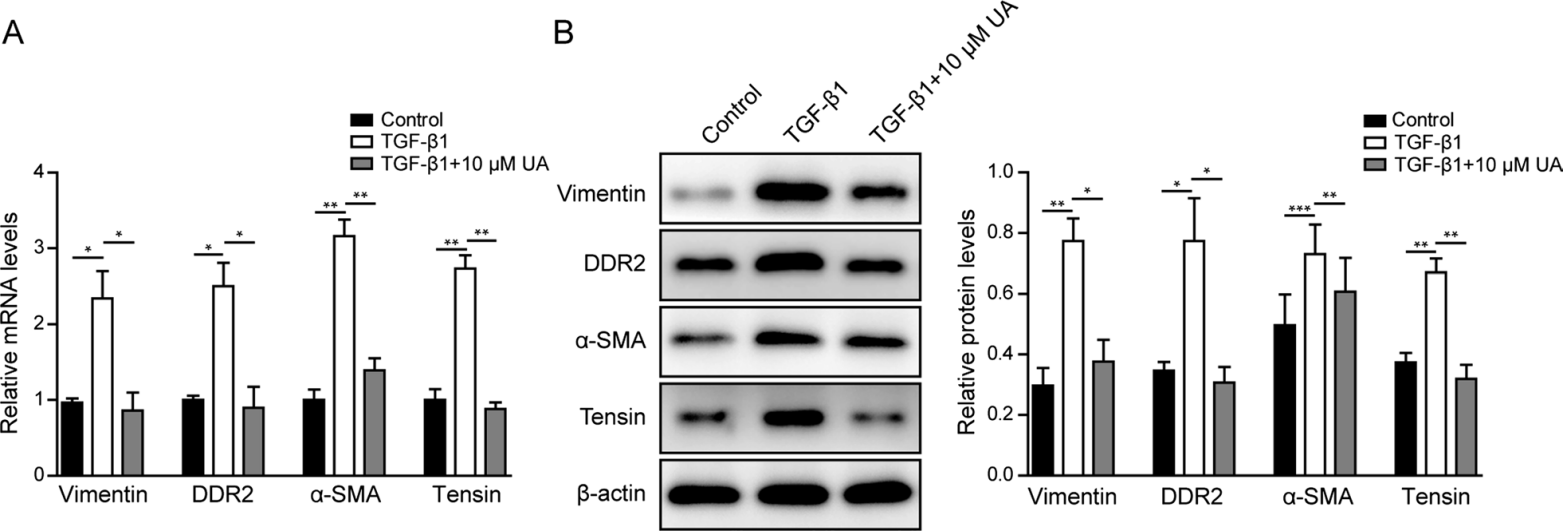
UA treatment repressed TGF-β1-induced transformation of cardiac fibroblasts to myofibroblasts. **A** The mRNA and **B** protein levels of vimentin, DDR2, α-SMA and tensin in CFs after indicated treatments. β-actin was used as normalized control. N = 3, *P < 0.05, **P < 0.01, ***P < 0.001

In order to determine the mechanism of UA’s function in myofibroblast transformation, several markers were tested. The mRNA and protein levels of fibrosis markers including vimentin, DDR2, tensin and α-SMA were significantly increased upon TGF-β1 treatment in CFs compared to control group (Fig. 2A, B), indicating a transformation from cardiac fibroblasts into myofibroblasts. However, when 10 μM UA was added together with TGF-β1, the expression levels of these fibrosis markers were inhibited compared to the group of TGF-β1 treatment alone (Fig. 2A, B). These results demonstrated that UA treatment could obviously repress TGF-β1-induced transformation of cardiac fibroblasts to myofibroblasts.

### UA inhibited proliferation, migration and invasion of TGF-β1-stimulated CFs through activating Nrf2

**Fig. 3:**
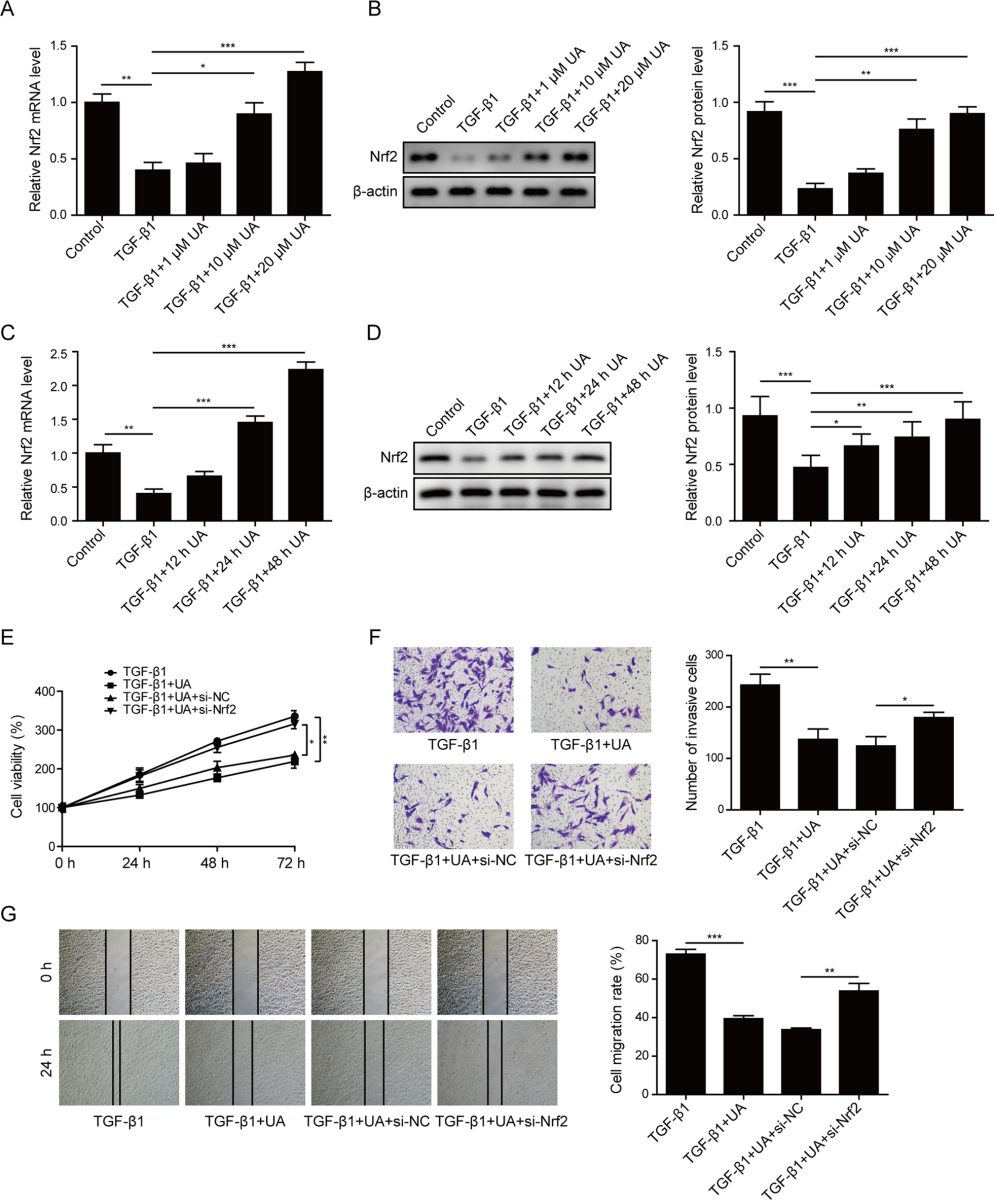
UA inhibited proliferation, migration and invasion of TGF-β1-stimulated CFs through activating Nrf2. **A** and C The mRNA level of Nrf2 in CFs after indicated treatments. **B** and **D** The protein level of Nrf2 in CFs after indicated treatments. β-actin was used as normalized control. **E** Cell viability was measured by MTT assay of CFs after indicated treatments. **F** Cell migration was measured by wound healing assay of CFs after indicated treatments. **G** Cell invasion ability was measured by transwell assay of CFs after indicated treatments. N = 3, *P < 0.05, **P < 0.01, ***P < 0.001

Nrf2 was reported as a key regulator for the CMT process. Our results showed that Nrf2 expression was significantly decreased in TGF-β1-treated CFs in both mRNA and protein levels compared to control group (Fig. 3A–D). When combined with TGF-β1 and UA treatments, the expression of Nrf2 was gradually recovered in a dose- and time-dependent manner as shown in Fig. 3A–D. To further identify role of Nrf2 in UA-mediated CMT inhibition, siRNA against Nrf2 was applied to knock down its expression in CFs. As shown in Fig. 3E, UA treatment impaired proliferation of TGF-β1-treated CFs, which was then recovered by knocking down Nrf2 expression. Similarly, the repressed migration and invasion abilities by UA treatment in TGF-β1-stimulated CFs were also rescued by inhibition of Nrf2 level (Fig. 3F–G). Taken together, these data suggested that Nrf2 was activated by UA treatment in TGF-β1-induced CFs and knocking down of Nrf2 could rescue the inhibition of UA on CMT process.

### Nrf2 pathway was responsible for UA-induced repression of CMT process in CFs with TGF-β1 treatment

**Fig. 4:**
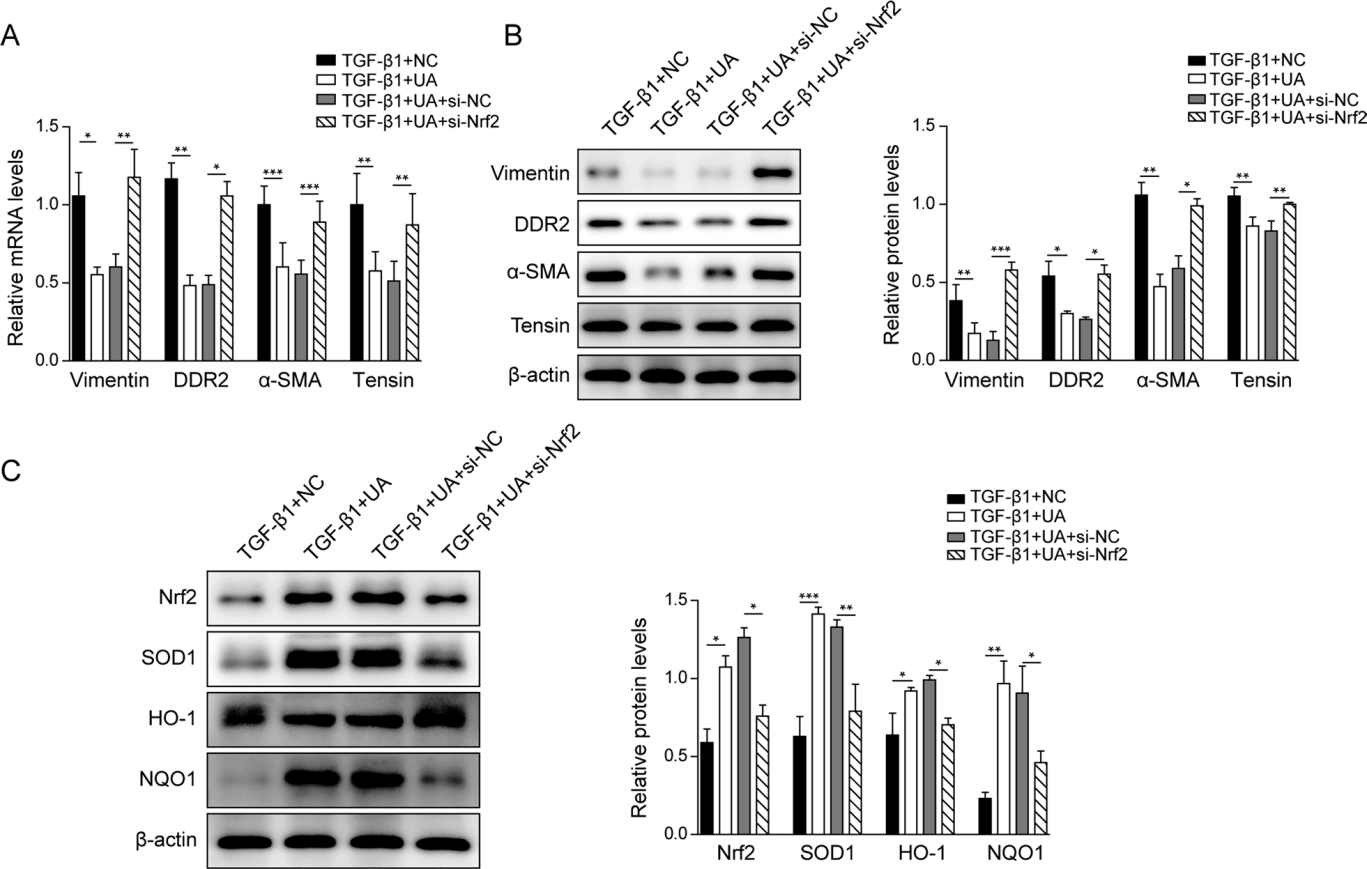
Nrf2 pathway was responsible for UA-induced repression of CMT process in CFs with TGF-β1 treatment. **A** The mRNA level and **B** the protein level of fibrosis markers including vimentin, DDR2, tensin and α-SMA in CFs after indicated treatments. **C** The protein levels of Nrf2, SOD1, HO-1 and NQO1 in CFs after indicated treatments. β-actin was used as normalized control. NC: negative control. N = 3, *P < 0.05, **P < 0.01, ***P < 0.001

Consistent with our finding that knocking down of Nrf2 could affect CMT process in CFs, the fibrosis markers were also changed upon manipulating Nrf2 expression. As shown in Fig. 4A, B, the decreased fibrosis markers including vimentin, DDR2, tensin and α-SMA by UA treatment were dramatically increased when Nrf2 expression was repressed. Moreover, the Nrf2 pathway-related markers were also evaluated. As shown in Fig. 4C, in TGF-β1-treated CFs, UA could induce the expression of Nrf2, as well as its downstream targets SOD1, HO-1 and NQO1, these effects were then reversed together with knocking down of Nrf2. In summary, these results demonstrated that Nrf2 pathway could regulate CMT process in UA-treated CFs.

### UA treatment inhibited myocardial fibrosis via Nrf2 pathway in vivo

**Fig. 5:**
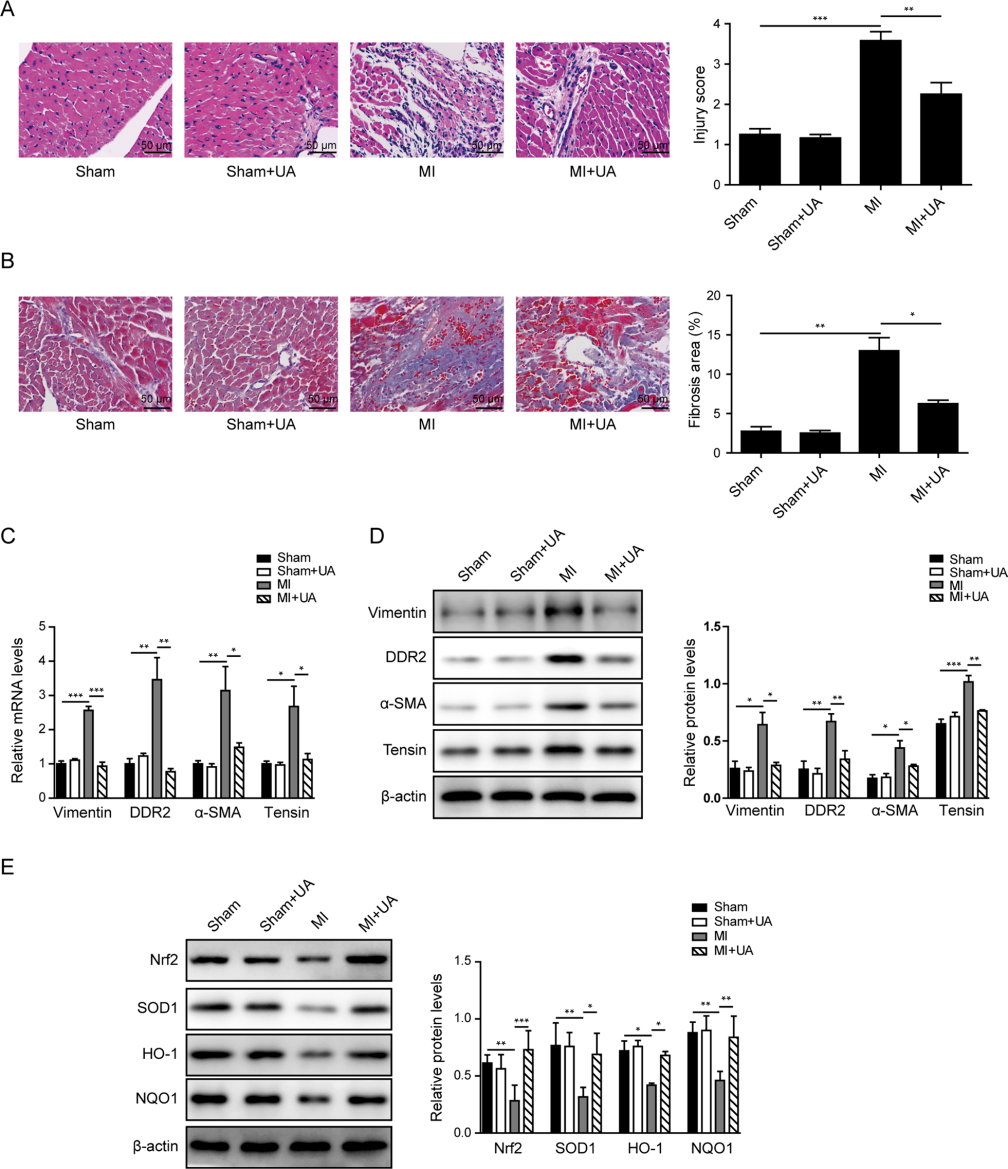
UA treatment inhibited myocardial fibrosis via Nrf2 pathway in vivo. **A** Represent images by H&E staining of heart tissues from indicated groups and statistic score of injury level. **B** Represent images by Masson’s trichrome staining of heart tissues from indicated groups and statistic results of the fibrosis area. **C** The mRNA levels and **D** protein levels of fibrosis markers including vimentin, DDR2, tensin and α-SMA in heart tissues from indicated groups. **E** The protein levels of Nrf2, SOD1, HO-1 and NQO1 in heart tissues from indicated groups. β-actin was used as normalized control. *P < 0.05, **P < 0.01, ***P < 0.001

To test our hypothesis that UA could repress myocardial fibrosis in vivo, we expanded the experiment to rat model for in vivo study. Rat MI model was established by surgery of LAD. Sham group without surgery was used as control. As shown in Fig. 5A, the H&E staining demonstrated an alleviated injury of myocardial tissues from rats in MI + UA group, compared to MI group without drug treatment. Meanwhile, the Masson staining also indicated that fibrosis level brought by MI surgery was significantly decreased when UA treatment was applied (Fig. 5B). Consistently, the expression levels of fibrosis markers including vimentin, DDR2, tensin and α-SMA in myocardial tissues were increased after MI surgery and then obviously reversed when UA was applied (Fig. 5C, D). Meanwhile, the Nrf2 and downstream targets of Nrf2 pathway were also affected in the myocardial tissues. As shown in Fig. 5E, the expression levels of Nrf2, SOD1, HO-1 and NQO1 were down-regulated after MI surgery, which were then significantly recovered when UA treatment was induced. Taken together, these data proved that UA treatment could block myocardial fibrosis via Nrf2 pathway in vivo.

## Discussion

It’s generally considered that transformation of cardiac fibroblasts to myofibroblasts (CMT) is an important event in the initiation of myocardial fibrosis (Zhou et al. 2018). The microenvironment associated with metabolic changes in tissues plays an important role in the CMT process, such as hyperglycemia, cellular oxidative stress and inflammatory cytokines dysregulation, which directly affects various molecular pathways related to cardiomyocyte function and structural damage (Savi et al. 2017).

It has been shown that gut microbiota plays important roles in the pathogenesis of cardiovascular diseases (Miele et al. 2015). The function of gut microbiota on cardiac fibrosis has been demonstrated by previous reports (Karbach et al. 2016; Organ et al. 2016). Urolithin A, derived from dietary polyphenols by microbiota has been linked to the beneficial effects related with high consumption of fruits and vegetables in humans (Vicinanza et al. 2013). Previous researches indicated the function of UA on inhibition of inflammation, proliferation and aging in different disease models (Singh et al. 2019; Zhang et al. 2019). However, the molecular targets and mechanisms of UA on pathophysiological processes are still unknown. In vivo UA treatment has been demonstrated to reduce the inflammatory state of cardiac tissues and prevent heart dysfunction in rat model of diabetes (Savi et al. 2017). The function of UA in myocardial fibrosis has not been explored yet. Our study for the first time demonstrated that UA treatment could inhibit the CMT process in TGF-β1-treated cardiac fibroblasts model by regulating cell proliferation, migration and invasion abilities in a dose- and time-dependent manners. Specifically, fibrosis markers altered significantly in response to UA treatment, including the inhibition of vimentin, DDR2, tensin and α-SMA, counteracting the effects of TGF-β1 stimulation. These results indicated a potential mechanism of UA in repressing TGF-β1-induced CMT process in CFs in vitro.

As a critical anti-oxidative gene, studies about the functions of Nrf2 on cardiovascular and metabolic diseases are increasing recently (Duan et al. 2017; Zhang et al. 2014). It has been reported that Nrf2 stimulation blocked TGF-β1-induced fibrotic genes upregulation in renal tubular epithelial cells (Yu et al. 2015). The pathophysiological consequences of Nrf2 activation is tightly related to the functional integrity of myocardial autophagy during cardiac remodeling (Qin et al. 2016). In our study, Nrf2 was shown to be repressed in TGF-β1-treated CFs. However, when UA treatment was applied together with TGF-β1, the level of Nrf2 was significantly recovered in dose- and time-dependent manners, as well as its downstream targets. To further determine if UA affected CMT process through regulating Nrf2 expression, we used Nrf2 specific siRNA to knock down its level in CFs and found that the inhibited abilities of cell proliferation, migration and invasion by UA treatment were enhanced by Nrf2 knocking down. Consistently, the expression of markers for fibrosis (vimentin, DDR2, tensin and α-SMA) and downstream targets of Nrf2 pathway (SOD1, HO-1 and NQO1) altered by UA treatment were rescued by knocking down Nrf2 level. These results demonstrated the critical role of Nrf2 in UA-mediated CMT process and explained the mechanism of UA’s function in mitigating cardiac fibrosis.

Moreover, our study also tested the UA’s function in vivo via rat MI model. We found that UA treatment could significantly alleviate the levels of tissue damage and fibrosis caused by MI surgery in rats’ hearts. However, the conclusion was limited due to the lack of zoomed out images showing the full LV section. Comprehensive comparison will be included in the future study. Consistent with the in vitro results, MI-induced myocardial fibrosis represented by the alteration of markers was significantly inhibited by UA administration. Meanwhile, the MI surgery inhibited Nrf2 pathway in cardiac tissues, which was then rescued by UA treatment. Overall, these results provided the evidences to demonstrate UA’s function in vivo.

## Conclusions

In summary, the present work demonstrated that UA could inhibit TGF-β1-induced CMT process in cardiac fibroblasts via upregulating Nrf2 expression. In vivo administration of UA could significantly improve myocardial fibrosis from myocardial infarction surgery in rat model. Regarding the current clinical exploration of UA on skeletal muscle function, iron metabolism and endurance performance, our study further explored the function and mechanism of UA in cardiac fibrosis and highlighted the application of UA in the clinical treatment of cardiovascular diseases in the future.

## Data Availability

All data generated or analyzed during this study are included in this published article.

## Abbreviations

MI: Myocardial infarction
CFs: Cardiac fibroblasts
CMT: Myofibroblast transformation
UA: Urolithin A
LAD: Left anterior descending coronary artery
ETs: Ellagitannins
Nrf2: Nuclear factor erythroid 2-related factor 2
AREs: Antioxidant response elements
HO1: Heme oxygenase-1
NQO1: NAD(P)H dehydrogenase [quinone] 1
DMEM: Dulbecco’s modified Eagle’s medium
FBS: Fetal bovine serum
qRT-PCR: Quantitative real-time PCR
MTT: 3-(4,5-Dimethylthiazol-2-yl)-2,5-diphenyl tetrazolium bromide
LV: Left ventricle
